# American Medical Association

**Published:** 1854-02

**Authors:** 


### AMERICAN MEDICAL ASSOCIATION.

The Seventh Annual Meeting of the American Medical Association will be held in the city of St. Louis on Tuesday, May, 2d, 1854.

The Secretaries of all Societies in the Association are requested to forward to the undersigned, correct lists of their respective delegations *as soon as they may be appointed*,—and it is *earnestly* desired by the Committee of Arrangements that the appointments be made at as early a period as possible.

The following are extracts from Art. 2d, of the Constitution:—

"Each local Society shall have the privilege of sending to the Association one Delegate for every ten of its regular resident members, and and for every additional fraction of more than half of this number. The Faculty of every regularly constituted Medical College or chartered School of Medicine shall have the privilege of sending two delegates. The professional staff of every chartered or municipal Hospital, containing an hundred inmates, or more, shall have the privilege of sending two delegates,—and every other permanently organized Medical Institution, of good standing, shall have the privilege of sending one delegate.

"Delegates representing the Medical staffs of the United States Army and Navy shall be appointed by the Chiefs of the Army and Navy Medical Bureaux. The number of delegates so appointed shall be, four from the Army Medical Officers, and a like number from the Navy Medical Officers."

The latter clause, in relation to delegates from the Army and Navy, was adopted as an amendment to Article 2nd of the Constitution, at the last annual meeting of the Association held in New York, in May, 1853.

E. S. LEMOINE. St. Louis.

*One of the Secretaries.*

N. B. The Medical Press of the United States is respectfully requested to copy the foregoing.

